# Fire-embracers possess higher shoot flammability, lower litter flammability and a more acquisitive strategy than fire-tolerators in *Pinus yunnanensis*

**DOI:** 10.1016/j.pld.2026.03.013

**Published:** 2026-04-10

**Authors:** Dachuan Dai, Shuting Li, Ying Liu, Zihan Zhang, Lihua Tu, Congde Huang, Shixing Zhou, Junxi Hu, Yang Liu, Xinhua He, Juli G. Pausas, Xinglei Cui

**Affiliations:** a College of Forestry, Sichuan Agricultural University, Chengdu 611130, China; b Sichuan Mt. Emei Forest Ecosystem National Observation and Research Station, Emei 614200, China; c Sichuan Academy of Forestry, Chengdu 610081, China; d School of Biological Sciences, University of Western Australia, Perth 6009, WA, Australia; e Department of Land, Air and Water Resources, University of California at Davis, Davis, CA 95616, USA; f CIDE-CSIC, Consejo Superior de Investigaciones Científicas, Montcada, Valencia 46113, Spain

**Keywords:** Wildfire, Plant flammability, Fire-adaptation, Survival strategy, Plant economics spectrum, *Pinus yunnanensis*

## Abstract

Plants in fire-prone environments have evolved traits to survive recurrent fires. Some traits enable plants to survive under crown fires (fire-embracer strategy), while others support persistence under surface fires (fire-tolerator strategy). Although plant fire survival strategies have been extensively studied, the manner in which they interact with other complementary survival strategies, such as flammability strategy and plant economic strategy, to facilitate plant coexistence with wildfires remain poorly investigated. *Pinus yunnanensis*, a fire-adapted species native to southwest China, has diversified into three distinct varieties: var. *yunnanensis*, var. *pygmaea* and var. *tenuifolia*. We examined the variations in fire-adaptations traits, shoot flammability, litter flammability, and plant economics traits across 21 populations from their three varieties. The relationships across these traits were also investigated to better understand their interplay. We found that *P*. *yunnanensis* has developed distinct fire survival strategies. Specifically, var. *pygmaea* survives wildfire as fire-embracers due to its high serotiny level, low crown height and thin bark; var. *tenuifolia* survives wildfire as fire-tolerators characterized by low serotiny level, high crown and thick bark, while the original var. *yunnanensis* exhibits the intermediate values in these fire-adaptions. Populations exhibiting fire-embracer strategy possess higher shoot flammability, lower litter flammability and a more acquisitive strategy compared to those with fire-tolerator strategy. Our study elucidated the divergent evolutionary trajectories among *P*. *yunnanensis* varieties to survive wildfires, and uncovered how the interplay among flammability attributes, fire-adaptations and economic traits facilitated the coexistence of *P*. *yunnanensis* with wildfires.

## Highlights

• Revealed coupling of fire adaptation strategy, flammability, and plant economics.
• Fire-embracer (var. *pygmaea*) employs high shoot, low litter flammability, acquisitive life.
• Fire-tolerator (var. *tenuifolia*) employs low shoot, high litter flammability, conservative life.

## 1 Introduction

Fire is a natural disturbance and evolutionary pressure that has shaped the evolutionary processes of many plant species, since the first terrestrial plant communities (about 420 million years ago; Glasspool et al., 2004; Pausas and Keeley, 2009; Keeley et al., 2011; Archibald et al., 2018; McLauchlan et al., 2020). Species in fire-prone environments usually have developed fire-adapted traits, such as thick bark, serotiny and high crown, to survive fires (Archibald et al., 2018; Lamont et al., 2020; Keeley and Pausas, 2022). Serotiny, which refers to the prolonged storage of seeds in cones or fruits until the heat of a fire trigger the release of seeds, can confer benefits to plants when fire return intervals fall between the age to reproductive maturity and the plant life span (Lamont, 1991; Lamont et al., 2020). The level of serotiny (the percentage of closed cones in relation to total cones) can vary greatly within species and is usually related to the frequency of crown fires (Radeloff et al., 2004; Hernández-Serrano et al., 2013; Pausas, 2015a; Battersby et al., 2017). For example, a study of two iconic Mediterranean pine species (*Pinus halepensis* and *P*. *pinaster*) showed that populations living under high crown-fire recurrence regimes had a higher serotiny level than those populations where the recurrence of crown-fires was low (Hernández-Serrano et al., 2013). A study of serotiny in *P*. *banksiana* showed that serotiny was higher in pine forests that exhibited stand-replacing fires, and lower in savannas where more frequent but less intense ground fires occurred (Radeloff et al., 2004). Insulating thick bark and self-pruning are also important fire-adaptations (Schwilk and Ackerly, 2001). Many pines have developed thick insulating barks, such as *P*. *pinea* (Madrigal et al., 2019) and self-pruning, such as *P*. *arizonica* and *P*. *montezumae* (Rodríguez-Trejo and Fulé, 2003; He et al., 2012) to survive under surface fire regimes. Insulating bark can protect the inner living tissues of the stem from damage by fire, thus increasing the survival chance of plants (Pausas, 2015b; Ne'eman and Arianoutsou, 2021), while self-pruning can generate a fuel gap between the understory and crown, reducing the chance of surface fires to climb up to the trees' canopies (Ne'eman and Arianoutsou, 2021).

Based on fire-adapted traits, pine species can be categorised into three fire syndromes: fire-tolerators, fire-embracers, and fire-avoiders (Keeley, 2011; Pausas, 2015a). Fire-avoiders generally lack specific fire adaptations and are restricted to habitats where fire is rare. In contrast, species in fire-prone environments primarily exhibit either a fire-tolerator or fire-embracer strategy. Fire-tolerators can survive frequent surface fires by developing traits such as self-pruning, high crowns and thick bark, whereas fire-embracers can rapidly recover their populations after crown fires via their high regeneration ability, conferred by traits such as serotiny and epicormic buds (Pausas, 2015a). Most pine species are characterized by a single strategy, while a few species can be either a fire-tolerator or fire-embracer, such as *Pinus pinaster*, which shows great intraspecific variation in serotiny level and bark thickness (Tapias et al., 2004). The diversity of fire survival strategies among fire-adapted species has been thoroughly investigated. Nevertheless, the relationship between fire survival strategies and plant flammability attributes, as well as the interplay with other survival strategies, such as plant economic strategy, has been scarcely reported.

Plant flammability, which describes the capacity of plants to ignite and sustain a flame, is affected by many functional traits. The flammability of plants varies widely among (Calitz et al., 2015; Wyse et al., 2016; Cui et al., 2020), even within species (Pausas et al., 2012; Battersby et al., 2017). Plant flammability can influence the survival of plant species in fire-prone environment (Gill and Zylstra, 2005; Pausas et al., 2017). For example, high shoot flammability can confer benefits to plant species in fire-prone ecosystems by increasing the mortality rate of neighboring competitor species and creating more space and richer nutrition for their progeny (Mutch, 1970; Pausas et al., 2012; Cui et al., 2020), although this hypothesis remains under debated (Snyder, 1984; Bond and Midgley, 1995; Gagnon et al., 2010; Midgley, 2013; Bowman et al., 2014). Pausas (2015a) suggested that the litter flammability of fire-tolerators is higher, while litter flammability of fire-embracers is lower, which may result in fire-tolerators experienced frequent surface fires while fire-embracers usually experience less surface fires. Plants living in fire-prone environments are likely to have evolved a range of flammability strategies, which may provide advantages to those plants subjected to varying fire regimes (Pausas et al., 2017).

To comprehend the survival strategy of plants in fire-prone environments, plant economics spectrum (PES), which entails the trade-offs between resource acquisition and conservation strategies occurring across various plant organs, such as leaves, stems, and roots (Reich, 2014; Kurze et al., 2021; Li et al., 2022), is also an important framework to investigate. The PES offers a conceptual framework for understanding how plants allocate limited resources to maximize their fitness under various environmental conditions (Reich et al., 1997; Wright et al., 2004; Reich, 2014). Along the PES, species employing an acquisitive strategy invest in rapid growth and high reproductive effort, with high leaf nutrient concentrations, gas exchange rates, and low leaf mass per area, leading to quick decomposition and nutrient cycling, but short lifespan and flimsy, disposable tissues (Reich et al., 1992, 1997; Wright et al., 2004). In contrast, plants with a conservative strategy prioritize resource conservation and longevity, with low gas exchange rates, leaf nutrient concentrations, and high leaf mass per area, which endow them with more robust tissues capable of enduring stresses like water scarcity and nutrient depletion (Freschet et al., 2010; Reich, 2014; Kunstler et al., 2016). Michelaki et al. (2020) demonstrated that acquisitive leaves with low LMA and high nutrient concentrations ignite quickly and sustain fire longer, whereas conservative leaves with high LMA and lower nutrient levels burn more completely and release more heat. Therefore, it is plausible to speculate that there may also be correlations between fire adaptation strategies and resource trade-off strategies, although this remains unstudied.

**Fig. 1:**
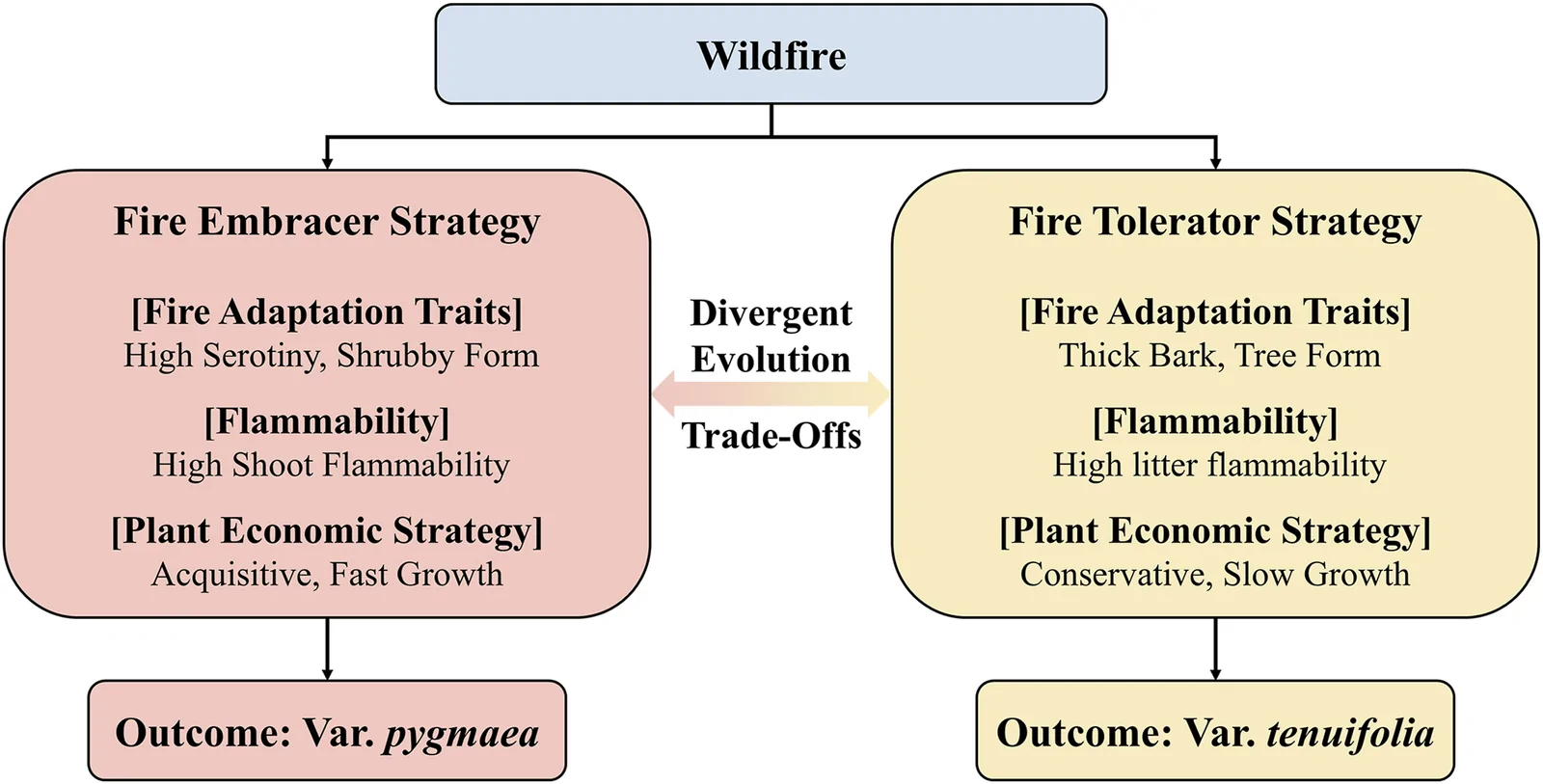
Conceptual framework of the divergent fire survival strategies in *Pinus yunnanensis*. The diagram illustrates how contrasting fire regimes (crown vs. surface fires) act as evolutionary drivers, selecting for distinct combinations of fire adaptation, flammability, and economic traits, thereby leading to the differentiation between fire-embracer and fire-tolerator strategies.

*Pinus yunnanensis* is an endemic species native to southwest China, where has a subtropical monsoon climate with alternating warm dry season and hot wet seasons, creating conditions that favour wildfires (Wu et al., 2020). Charcoal evidence has showed that this region has experienced frequent wildfires since late Permian (Shao et al., 2012; Yan et al., 2019). Due to the fire-proneness of this region, many species there, including *P*. *yunnanensis*, have developed fire adaptations (Su et al., 2015; Chen et al., 2020; Pausas et al., 2021). Considerable morphological and genetic variations exist within *P*. *yunnanensis* (Sun et al., 2020), leading to the recognition of three distinct varieties within *P*. *yunnanensis*: var. *yunnanensis*, var. *pygmaea* and var. *tenuifolia*. Var. *yunnanensis* and var. *tenuifolia* are trees, while var. *pygmaea* typically manifests as a multi-stemmed shrub, reaching a maximum height of only 2 m. Fire-adapted traits, including serotiny, thick bark, self-pruning, also vary greatly within species (He et al., 2012; Pausas, 2015b; Lamont et al., 2020). Therefore, we hypothesize that *P*. *yunnanensis* is diversifying to occupy different niches defined by different fire regimes, and thus we predict that 1) different varieties of *P*. *yunnanensis* show different traits and fire strategies (fire-embracers, fire-tolerators); 2) the shoot flammability of fire-embracers is higher than fire-tolerators, while litter flammability of fire-embracers is lower than fire-tolerators in *P*. *yunnanensis*; 3) Fire-embracers possess a more acquisitive strategy than fire-tolerators in *P*. *yunnanensis*. A conceptual framework illustrating the formation of these distinct adaptive syndromes is presented in Fig. 1. To examine our hypothesis, we investigated the intraspecific variation in fire-adaptations, economics traits, shoot flammability and litter flammability within *P*. *yunnanensis*, and investigated the relationships among these traits.

## 2 Materials and methods

### 2.1 Samples collection

**Fig. 2:**
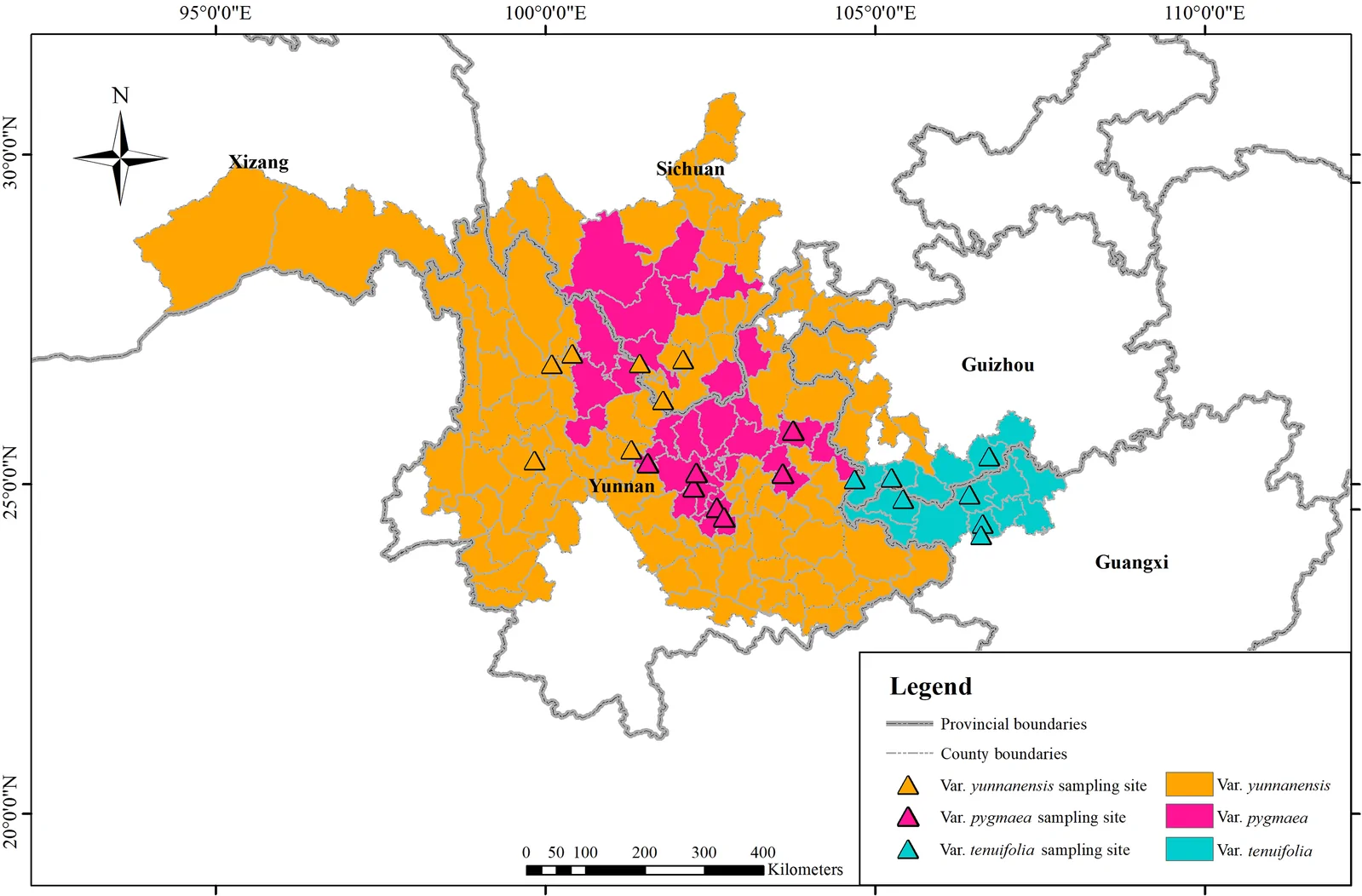
The distribution of sampling sites. Different colours represent the primary distribution ranges of different varieties, and the coloured triangles represent sampling sites. Var. *yunnanensis* is distributed across almost the entire distribution area of *P*. *yunnanensis*, including the distribution ranges of var. *pygmaea* and var. *tenuifolia*. Distribution data were from the studies of Jin and Peng (2004), Chen et al. (2012), and The First National Forest and Grassland Fire Risk Survey of the People's Republic of China.

We collected samples of *Pinus* *yunnanensis* from a total of 21 sites, with 7 sampling sites designated for each variety (Fig. 2). On each sampling site, 6 mature and healthy individuals were randomly selected, and a 70 cm terminal shoot was collected from each tree for the assessment of shoot flammability. Additionally, twigs and leaves were gathered from the same individual for the determination of physicochemical characteristics. Shoot samples were immediately stored at 4 °C to prevent desiccation. Twigs and leaves samples were weighed and then sealed in plastic bags.

For litter collection, we obtained fresh samples from beneath each tree by gathering dead, undecomposed material from the ground or by lightly shaking branches to release litter. The goal was to acquire leaf material in its natural state as dropped by the tree (Cornwell et al., 2015). These samples were then air-dried for 4–8 weeks at 21 °C in a temperature-controlled laboratory to simulate the very low moisture conditions typical of periods with high fire risk.

### 2.2 Environmental data acquisition

To characterize the ecological context, we extracted Mean Annual Temperature (MAT) and Mean Annual Precipitation (MAP) from the WorldClim v.2 database (Fick and Hijmans, 2017) and calculated a Fire Activity index following Pausas and Ribeiro (2013) for all 21 study sites. The fire activity index was derived from MODIS fire incidence data to reflect wildfire frequency and extent. Detailed protocols for these environmental data extractions are described in our previous study (Dai et al., 2025).

### 2.3 Traits measurement

Crown height, defined as the vertical distance from the ground to the base of the living crown, was measured using a laser rangefinder and leveling rod from 6 mature individuals per site. For var*. pygmaea*, which typically exhibits a shrubby growth form branching from the base, crown height was recorded as 0 m to reflect the absence of a gap between surface and canopy fuels.

Bark thickness was measured following Pausas (2015b), referring to total bark thickness (inner bark plus outer bark). A bark puncher was used to extract a woody core sample at breast height (1.3 m) for var. *yunnanensis* and var. *tenuifolia*, and at 10 cm above the ground for var. *pygmaea*. The punched cores were promptly measured using a vernier caliper.

Serotiny level was evaluated by observing the degree of cone opening on selected individuals. For var. *yunnanensis* and var. *tenuifolia*, due to the tall crown height that limited whole-tree observation, we randomly selected branches from the upper, middle, and lower parts to record the proportion of open and closed mature cones. For var. *pygmaea*, mature cones were documented across the whole plant. Serotiny level was calculated based on the number of open versus closed mature cones recorded for each individual.

Relative moisture content (RMC) was calculated as RMC (%) = (m_0_–m_2_)/(m_1_–m_2_) × 100%, where m_0_ is the fresh mass, m_1_ the saturated mass (after 24 h rehydration), and m_2_ the dry mass (after oven-drying at 105 °C to constant weight).

Leaf length and leaf area were assessed by scanning leaves at 300 DPI using a scanner (HP Laser MFP 136w, HP Inc., Palo Alto, CA, USA). Scanned Images were analyzed in ImageJ 1.53a (NIH, Bethesda, MD, USA) to measure leaf length and leaf area. Leaf mass per area was calculated as leaf dry mass divided by leaf area (g m^−2^).

Calorific value was determined by drying samples to constant weight at 105 °C, grinding, sieving through a 15-mesh screen, and measuring calorific value with an automatic calorimeter (ZDHW-9, Jiangsu Tianyuan Testing Equipment Co., Ltd., China).

Ash content was measured by combusting oven-dried samples in a muffle furnace at 650 °C for 4 h and cooling for 30 min. Ash content (%) = ash mass/total oven-dried mass × 100%.

Tannin content was determined by extracting ground plant material with dimethylformamide, followed by filtration and complexation with ferric ammonium citrate. The absorbance of the resulting complex was measured at 525 nm, and tannin concentrations were expressed as tannic acid equivalents using a standard calibration curve.

Crude fat content was determined by drying samples at 105 °C, grinding to 40 mesh, and extracting with anhydrous diethyl ether in a Soxhlet extractor for 8–12 h. Extracts were evaporated and dried at 105 °C for 2 h. Crude fat content (%) = fat mass/total oven-dried mass × 100%.

Cellulose content was obtained by refluxing ground samples (G_0_) with nitric acid–ethanol (5: 2, v: v) at 100 °C, repeated until residues turned white. The residue was sequentially washed with nitric acid-ethanol solution, hot distilled water, and anhydrous ethanol. After vacuum filtration, the residue was oven-dried at 105 °C to constant weight (G_1_), followed by incineration at 500 °C until constant mass (G_2_) was attained. Cellulose content (%) = [(G_1_–G_2_)/G_0_] × 100%.

Leaf nitrogen content per unit mass (*N*_mass_) was measured using an Element Analyzer (VARIO EL III Element Analyzer, Elementar, Germany). Leaf phosphorus content per unit mass (*P*_mass_) was measured using the molydate/ascorbic acid method and a continuous flow analyzer (SKALAR SAN++, Netherlands) after H_2_SO_4_–HClO_4_ (4:1, v:v) digestion.

Gas exchange traits were measured with a LI-6800 portable photosynthesis system (LI-COR, Lincoln, NE, USA) between 9:00 and 11:30 a.m. The reference CO_2_ concentration was maintained at 400 μmol mol^−1^, the leaf chamber temperature at 25 °C, the relative humidity at 60%, and the photosynthetic active radiation at 1600 μmol m^−2^ s^−1^. Branches were cut from the canopy and immediately recut underwater to prevent embolism formation (Yoder et al., 1994). Photosynthetic rate (A_area_) and respiration rates (R_area_) were converted to their mass-based equivalents (A_mass_, R_mass_) using the leaf mass per area (LMA) based on the varieties' average values.

Age at sexual maturity has been shown to depend on the fire regime in other pines (Guiote and Pausas, 2023). In this study, the data were obtained from published literature (Chen and An, 1993; Fang et al., 2015; Yang et al., 2015) and through consultation with field experts.

Litter structural traits were assessed using subsamples taken from each air-dried litter sample prior to the flammability experiment. We measured the packing ratio (litter particle volume/litterbed volume), litterbed density (litter particle mass/litterbed volume), surface area: litter particle volume (litter particle surface area/litter particle volume), surface area: litterbed volume (litter particle surface area/litterbed volume) and litter tissue density (litter particle mass/litter particle volume). Litter particle volume was measured using the water-displacement method (Pérez-Harguindeguy et al., 2013), litter particle surface area was obtained via scanned images, and litter particle mass was determined by weighing after drying. Litterbed volume corresponded to the fixed container volume.

### 2.4 Fire experiment

Shoot flammability was measured following the methods described by Jaureguiberry et al. (2011) and Wyse et al. (2016), ignition time (IT), maximum temperature (MT), burning time (BT), and burnt biomass (BB), were measured by burning at least six 70 cm length shoot samples (Cui et al., 2020). Before burning, all of the shoot samples were placed in a constant temperature and humidity growth chamber with 25 °C and 65% humidity for 24h to eliminate possible errors between different plant samples due to different sampling times and locations (according to Pausas et al., 2012). Ignition time refers to the time it takes for the sample to ignite within the first 20 s of ignition from a torch (the time that ignites the flame), representing the sample's ignitibility. Maximum temperature of the burning sample recorded on an infrared laser thermometer after the blowtorch was turned off and represents combustibility. Burning time is how long the sample burns after turning off the blowtorch and represents sustainability. Burnt biomass is the percentage of combusted biomass after the flame goes out and represents consumability.

Litter flammability was tested using the air-dried litter samples placed on a 50 cm × 40 cm × 3 cm steel mesh tray inclined at 15°, to simulate the slope conditions observed at the sampling site. All litter samples were held constant to a mass of 100g, while the height of the litterbed varied accordingly. The litter samples were evenly dropped into the tray and pressed the litter surface very gently to assume a spontaneous configuration, at a similar density to the observed density in the sampling sites (de Magalhães and Schwilk, 2021). After placing the samples, the blowtorch was turned on for a maximum of 20 s, starting from the central point of the tray to ignite the litter samples. We measured six different flammability traits: ignition time, maximum temperature, burning time and burnt biomass, the measurement method is consistent with the shoot flammability. Maximum flame height (MFH, cm) estimated from video recordings captured during the experimental process: flame height was determined through visual assessment every 1 s to the nearest cm using a graduated ruler as a landmark. Rate of spread (ROS, cm s^−1^) was calculated based on the time it took for the flame to reach the edge of the tray during the experiment. We acknowledge that the Rate of spread is indicative and cannot be directly compared to values measured in real wildfire situations. This limitation arises from the small size of our samples, and the fire propagation cannot reach a stable state under the experimental conditions (see McAlpine and Wakimoto, 1991).

### 2.5 Data analysis

Prior to statistical analysis, measurements from individuals within the same site were averaged to obtain site-level means. Consequently, the total sample size for the analysis was 21 (3 varieties × 7 sites). Trait differences among varieties were tested based on these site means using One-way ANOVA followed by Fisher's LSD (α = 0.05). Pearson correlations were also calculated using site means to examine trait relationships. Principal Component Analysis (PCA) was conducted to comprehensively assess the variations in fire adaptation, shoot flammability, litter flammability, and economic strategy among different varieties. The first principal component (PC1) for each PCA was defined as the respective index: fire adaptation index, shoot flammability index, litter flammability index, and plant economic index. All statistical analysis and plotting were performed by using R 4.2.3 and Origin 2025. Creating distribution maps was done using ArcGIS 10.2 (Esri, Redlands, CA, USA).

## 3 Results

### 3.1 *Pinusyunnanensis* has evolved to be either a fire-tolerator or fire-embracer

Significant differences were detected in serotiny level, bark thickness and crown height, among different varieties (Table 1). The serotiny level of var. *pygmaea* was found to be significantly higher (*P* < 0.05) when compared to both var. *yunnanensis* and var. *tenuifolia*. Var. *tenuifolia* exhibited a remarkable crown height, significantly surpassing var. *yunnanensis*. Var. *yunnanensis* and var. *tenuifolia* demonstrated a significantly thicker bark thickness than var. *pygmaea*.

**Table 1:** Fire adaptation traits, shoot flammability, litter flammability, physicochemical characteristics and functional traits of *Pinusyunnanensis*.

Traits	Var. *pygmaea*	Var. *yunnanensis*	Var. *tenuifolia*
**Fire adaptation traits**			
Crown height (m)	0.00 ± 0.00 c	4.92 ± 1.45b	9.19 ± 1.82a
Serotiny level (%)	69.67 ± 13.21a	10.30 ± 1.58b	7.78 ± 2.82b
Bark thickness (cm)	0.55 ± 0.09b	1.83 ± 0.37a	1.69 ± 0.21a
Fire adaptation index	2.18 ± 0.31a	−0.83 ± 0.36b	−1.35 ± 0.38c
**Shoot flammability**			
Ignition time (shoot) (s)	10.95 ± 2.12b	12.45 ± 2.52b	16.77 ± 0.95a
Burning time (shoot) (s)	21.51 ± 2.36a	3.24 ± 1.03b	3.65 ± 0.69b
Maximum temperature (shoot) (°C)	368.68 ± 45.49a	262.40 ± 13.65b	235.10 ± 13.25b
Burnt biomass (shoot) (%)	18.29 ± 4.19a	6.05 ± 1.69b	4.39 ± 0.79b
Shoot flammability index	2.35 ± 0.69a	−0.72 ± 0.47b	−1.63 ± 0.17c
**Litter flammability**			
Ignition time (litter) (s)	11.31 ± 0.91a	7.73 ± 1.30b	8.96 ± 1.16b
Burning time (litter) (s)	159.04 ± 16.18a	143.26 ± 17.84a	90.88 ± 6.81b
Maximum temperature (litter) (°C)	620.67 ± 23.27a	626.59 ± 13.95a	607.77 ± 29.68a
Burnt biomass (litter) (%)	98.00 ± 2.08a	98.71 ± 0.49a	98.86 ± 0.69a
Maximum flame height (litter) (cm)	56.00 ± 7.57b	58.43 ± 3.99b	81.29 ± 7.16a
Rate of spread (litter) (cm s^−1^)	0.28 ± 0.02b	0.31 ± 0.03b	0.42 ± 0.02a
Litter flammability index	−1.58 ± 0.67c	−0.54 ± 0.64b	2.12 ± 0.49a
**Physicochemical and morphological traits**			
Twig ash content (%)	1.31 ± 0.17b	1.90 ± 0.45a	2.33 ± 0.47a
Twig calorific value (kJ g^−1^)	21.76 ± 0.40a	21.71 ± 0.57a	21.11 ± 0.59b
Twig cellulose content (%)	11.34 ± 3.08a	11.56 ± 2.28a	11.45 ± 2.09a
Twig crude fat content (%)	6.12 ± 0.97a	6.53 ± 1.14a	5.32 ± 1.14a
Twig tannin content (g kg^−1^)	30.60 ± 3.02a	18.00 ± 4.57b	22.37 ± 5.80b
Twig relative moisture content (%)	73.91 ± 3.80b	83.96 ± 1.97a	83.84 ± 0.88a
Leaf ash content (%)	2.00 ± 0.21c	2.59 ± 0.38b	3.39 ± 0.29a
Leaf calorific value (kJ·g^−1^)	22.28 ± 0.31a	21.64 ± 0.35b	21.29 ± 0.46b
Leaf cellulose content (%)	9.12 ± 0.94a	9.59 ± 1.59a	9.07 ± 0.63a
Leaf crude fat content (%)	6.30 ± 0.77a	6.08 ± 0.89a	6.71 ± 1.25a
Leaf tannin content (g kg^−1^)	48.39 ± 8.31a	25.32 ± 3.87b	29.28 ± 3.91b
Leaf relative moisture content (%)	71.83 ± 2.45b	84.18 ± 2.29a	82.47 ± 3.01a
Leaf length (cm)	18.06 ± 1.13c	21.94 ± 0.61b	24.54 ± 0.31a
Leaf area (cm^2^)	1.40 ± 0.20b	2.00 ± 0.29a	1.58 ± 0.18b
**Litter structural traits**			
Litter tissue density (mg cm^−3^)	0.46 ± 0.04a	0.45 ± 0.07a	0.49 ± 0.02a
Litterbed density (mg cm^−3^)	39.90 ± 1.46a	29.89 ± 1.06b	31.69 ± 2.22b
Packing ratio (cm^3^ cm^−3^)	8.66 ± 0.64a	6.82 ± 1.12b	6.44 ± 0.72b
Surface area: litter particle volume (cm^2^ cm^−3^)	17.52 ± 0.85b	19.92 ± 3.38a	16.10 ± 1.16b
Surface area: litterbed volume (cm^2^ cm^−3^)	1.51 ± 0.08a	1.33 ± 0.05b	1.03 ± 0.06c
**Plant economic traits**			
Leaf mass per area (g m^−2^)	250.74 ± 15.15c	266.23 ± 5.41b	290.37 ± 8.15a
Leaf nitrogen content (mg g^−1^)	10.54 ± 1.73a	11.00 ± 0.91a	10.40 ± 0.81a
Leaf phosphorus content (mg g^−1^)	1.02 ± 0.30a	1.03 ± 0.02a	0.93 ± 0.02a
Photosynthetic rate (μmol g^−1^ s^−1^)	0.05 ± 0.01a	0.03 ± 0.00b	0.02 ± 0.00c
Respiration rate (μmol g^−1^ s^−1^)	0.01 ± 0.00a	0.01 ± 0.00b	0.01 ± 0.00b
Age at sexual maturity∗ (year)	5	10	11
Plant economic index	−2.18 ± 0.55c	0.46 ± 0.34b	1.73 ± 0.46a

Note: Populations with the same letter code are not significantly different, based on Fisher's Least Significant Difference (*P* < 0.05). The indices (Fire adaptation index, Shoot/Litter flammability index, and Plant economic index) represent the scores of the first principal component (PC1) derived from Principal Component Analysis (PCA) performed on the respective group of traits, serving as integrated metrics for each functional category. ∗ Data for ‘age at sexual maturity’ were retrieved from other research sources (Chen and An, 1993; Fang et al., 2015; Yang et al., 2015).

**Fig. 3:**
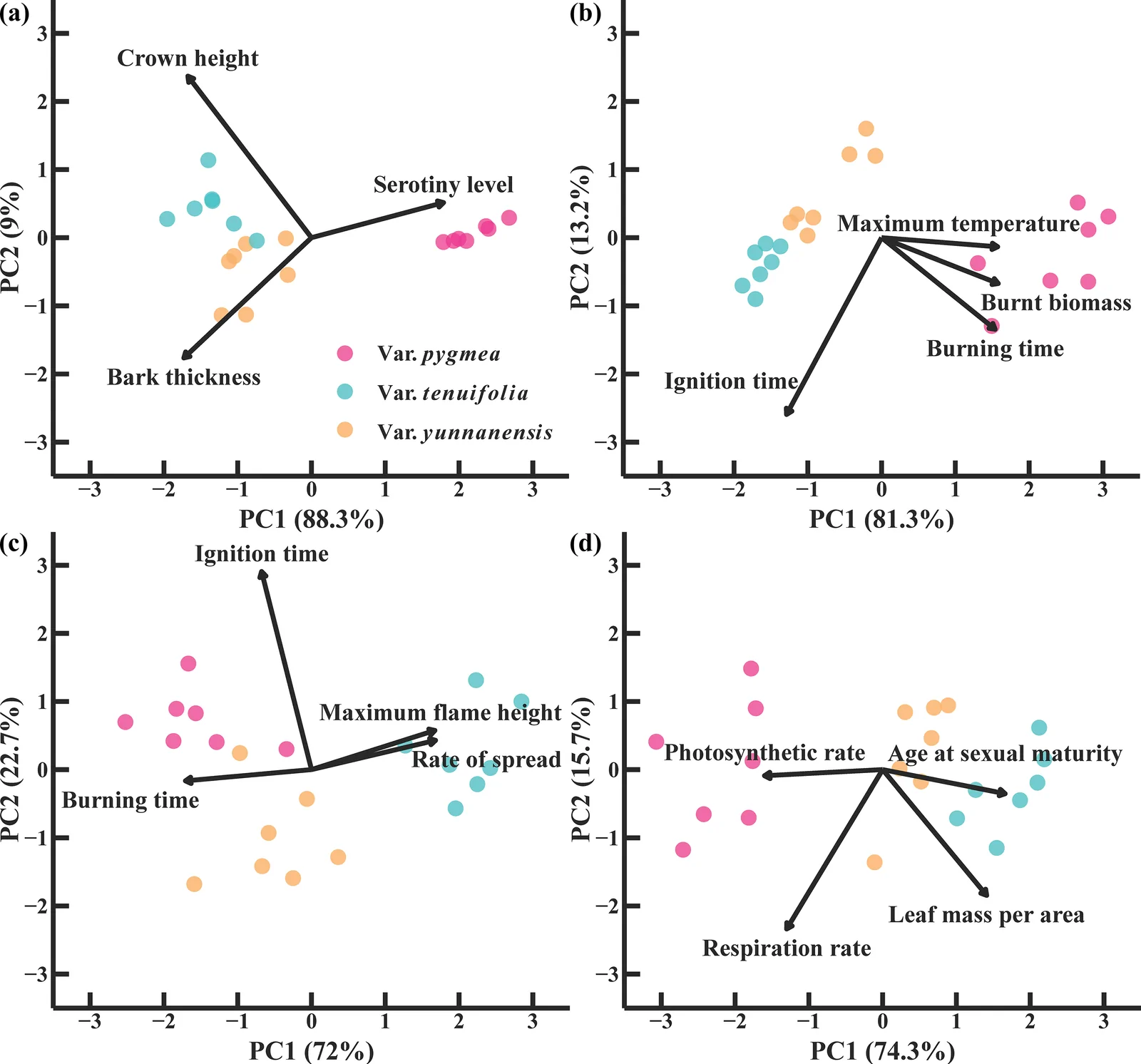
Principal component analysis (PCA) among three varieties, showing variation in (a) fire adaptation traits; (b) shoot flammability; (c) litter flammability; (d) plant economics traits. Points indicate the different sampling site, point colour indicate the variety.

Principal Component Analysis (PCA) using crown height, serotiny level, and bark thickness as variables explained 88.3% and 9.0% of the total variation with the first two principal axes, respectively (Fig. 3a). The first principal component (PC1) was defined as the fire adaptation index, which captures the variation in fire adaptation traits, with higher values indicating a stronger tendency towards fire embracing and lower values reflecting greater fire tolerance. Specifically, var. *pygmaea* displayed the significantly highest fire adaptation index than other varieties, indicating its high serotiny level and robust post-fire regenerative ability. Conversely, var. *tenuifolia* exhibited the significantly lowest fire adaptation index, suggesting a profile characterized by thick bark, high crown height, and low serotiny level (Table 1). Furthermore, environmental analysis revealed that the mean annual precipitation and fire activity index did not differ significantly among the habitats of the three varieties, although var. *tenuifolia* inhabits significantly warmer sites (Table S1). The consistently high fire activity index across all groups confirms that they face similar wildfire pressures, suggesting that the observed trait divergence is likely a response to specific fire regimes (e.g., surface vs. crown fires) rather than broad climatic or fire frequency gradients. These results suggested that var. *pygmaea* tends to embrace fire, showcasing a strategy of “fire-embracer” with elevated serotiny levels; var. *tenuifolia* appears to be more resilient to wildfires, adopting a “fire-tolerator” strategy with its combination of thick bark, high crown, and lower serotiny levels; while var. *yunnanensis* displays a “generalized strategy”, characterized by thick bark for surface fire resistance but possessing intermediate crown height and serotiny levels.

### 3.2 Fire-embracers exhibit higher shoot flammability than fire-tolerators in *Pinusyunnanensis*

The shoot flammability components varied significantly among the three varieties of *Pinus* *yunnanensis* (Table 1). Var. *pygmaea* exhibited the longest burning time, which was substantially greater than those of var. *yunnanensis* and var. *tenuifolia*. The maximum temperature also differed markedly, with var. *pygmaea* significantly higher than var. *yunnanensis* and var. *tenuifolia*. Furthermore, var. *pygmaea* demonstrated the greatest biomass consumption percentage, far exceeding those of var. *yunnanensis* and var. *tenuifolia*. While for ignition time, var. *tenuifolia* was significantly longer than those of var. *pygmaea* and var. *yunnanensis*.

PCA using four shoot flammability components as variables yielded the first two principal axes accounted for 81.3% and 13.2% of the total variance, respectively (Fig. 3b). These results indicate that PC1 serves as a robust composite measure, effectively capturing the key aspects of shoot flammability, including burning time, maximum temperature, burnt biomass, and ignition time. The PC1 score was defined as the shoot flammability index, with higher values reflecting stronger shoot flammability. Var. *pygmaea* received the significantly highest shoot flammability index, while var. *tenuifolia* had the lowest, indicating the highest and lowest shoot flammability, respectively, among the three varieties (Table 1).

To discern the factors influencing the variation in shoot flammability among varieties, we measured traits that potentially affect shoot flammability and analysed the relationship between shoot flammability components and these traits (Table 2). Overall, var. *pygmaea* exhibited higher shoot flammability, which may be ascribed to its increased twig calorific value, leaf calorific value, twig tannin content, and leaf tannin content. Conversely, var. *tenuifolia* exhibited lower shoot flammability, associated with its higher twig relative moisture content, leaf relative moisture content, twig ash content, leaf ash content, leaf length, and leaf mass per area.

**Table 2:** Correlation coefficients for the shoot flammability and functional traits.

	Ignition time	Burning time	Max temperature	Burnt biomass	Shoot flammability index
Twig ash content	**0.52**	**−0.67**	**−0.64**	**−0.66**	**−0.69**
Twig calorific value	**−0.51**	0.27	0.35	0.41	0.42
Twig cellulose content	0.06	−0.01	−0.17	−0.14	−0.11
Twig crude fat content	−0.39	0.08	0.15	0.29	0.25
Twig tannin content	−0.11	**0.72**	**0.67**	**0.58**	**0.59**
Twig relative moisture content	**0.47**	**−0.90**	**−0.80**	**−0.89**	**−0.86**
Leaf ash content	**0.72**	**−0.73**	**−0.80**	**−0.77**	**−0.84**
Leaf calorific value	**−0.54**	**0.71**	**0.53**	**0.72**	**0.70**
Leaf cellulose content	0.02	−0.12	−0.09	−0.21	−0.13
Leaf crude fat content	0.12	−0.04	−0.24	0.02	−0.11
Leaf tannin content	−0.37	**0.89**	**0.74**	**0.88**	**0.81**
Leaf relative moisture content	**0.46**	**−0.92**	**−0.78**	**−0.82**	**−0.84**
Leaf length	**0.68**	**−0.88**	**−0.89**	**−0.82**	**−0.91**
Leaf area	−0.16	**−0.56**	−0.35	−0.34	−0.32
Leaf mass per area	**0.71**	**−0.67**	**−0.74**	**−0.72**	**−0.78**

Note: Values where *P* < 0.05 are shown in bold.

### 3.3 Fire-embracers exhibit lower litter flammability than fire-tolerators in *Pinusyunnanensis*

Among the three varieties of *Pinus* *yunnanensis*, the litter of var. *pygmaea* showed the slowest ignition, significantly shorter than var. *yunnanensis* and var. *pygmaea*. Additionally, var. *tenuifolia* had the shortest burning duration, significantly lower than both var. *yunnanensis* and var. *pygmaea*. The maximum flame height was highest in var. *tenuifolia*, significantly greater than that of var. *pygmaea* and var. *yunnanensis*. Similarly, var. *tenuifolia* exhibited the fastest rate of spread, significantly outpacing var. *pygmaea* and var. *yunnanensis*. However, there were no significant differences among the varieties in maximum temperature and burnt biomass.

PCA was performed using four litter flammability components: ignition time, burning time, rate of spread, and maximum flame height (Fig. 3c). Maximum temperature and burnt biomass were excluded from the PCA because of their lack of significant differences among varieties. The first two principal axes accounted for 72.0% and 22.7% of the total variance, respectively. PC1 effectively captured the variation in litter flammability and was defined as the litter flammability index, with higher values indicating stronger litter flammability. Var. *tenuifolia* exhibited the highest litter flammability index, while var. *pygmaea* showed the lowest, reflecting the strongest and weakest litter flammability, respectively (Table 1).

In an effort to pinpoint the factors contributing to the variation in litter flammability among varieties, we measured traits that potentially affect litter flammability and analysed the relationship between litter flammability components and these traits (Table 3). Summarily, packing ratio, and surface area: litterbed volume showcased significant correlations with all four litter flammability components and litter flammability index. Var. *pygmaea* exhibited higher values of ignition time, burning time, and lower maximum flame height, rate of spread, due to its higher litterbed density, packing ratio, and surface area: litterbed volume. Conversely, var. *tenuifolia* tended to have lower litterbed density, packing ratio, and surface area: litterbed volume, thus, it displayed higher litter flammability.

**Table 3:** Correlation coefficients for the litter flammability and litter structural traits.

	Ignition time	Burning time	Rate of spread	Maximum flame height	Litter flammability index
Litter tissue density	0.07	−0.43	**0.48**	**0.53**	**0.47**
Litterbed density	**0.83**	0.42	−0.43	−0.32	**−0.50**
Packing ratio	**0.59**	**0.60**	**−0.63**	**−0.59**	**−0.68**
Surface area: litter particle volume	−0.22	0.32	−0.28	−0.29	−0.26
Surface area: litterbed volume	**0.48**	**0.88**	**−0.88**	**−0.85**	**−0.93**

Note: Values where *P* < 0.05 are shown in bold.

### 3.4 Fire-embracer tends to employ a more acquisitive strategy than fire-tolerator in *Pinusyunnanensis*

Significant differences in economic traits were observed among the three varieties (Table 1). Var. *tenuifolia* displayed the highest leaf mass per area (LMA), followed by var. *yunnanensis*, and var. *pygmaea*. Regarding gas exchange parameters, var. *pygmaea* exhibited the highest net photosynthetic rate and respiration rate, both of which were significantly greater than those of the other two varieties. In terms of nutrient content, leaf nitrogen (N_mass_) and phosphorus (P_mass_) content did not show significant differences among the varieties, which could be attributed to similar soil nutrient conditions in their respective habitats (Table S2). Literature data further suggest that var. *tenuifolia* takes the longest to reach sexual maturity, while var. *pygmaea* matures the earliest, with var. *yunnanensis* positioned between the other two.

For the plant economic strategy, PCA was performed using four key economic traits, as N_mass_ and P_mass_ did not show significant variation across the varieties. The first two principal components explained 74.3% and 15.7% of the total variance, respectively (Fig. 3d). PC1 was positively associated with LMA and age at sexual maturity, while gas exchange parameters (photosynthetic and respiration rates) contributed negative loadings. PC1 was defined as the plant economic index, where higher values indicated a more conservative strategy, while lower values suggest a tendency towards a more acquisitive strategy. Var. *tenuifolia* showed the significantly highest plant economic index, indicating conservative strategy characterized by high LMA, late sexual maturity, and lower gas exchange rates. On the opposite end, var. *pygmaea* had the significantly lowest plant economic index, signifying acquisitive strategy with lower LMA, earlier sexual maturity (i.e., precocity), and higher photosynthetic and respiration rates (Table 1).

### 3.5 Fire adaptation strategies are intrinsically coupled with flammability and plant economic strategies in *Pinus yunnanensis*

**Fig. 4:**
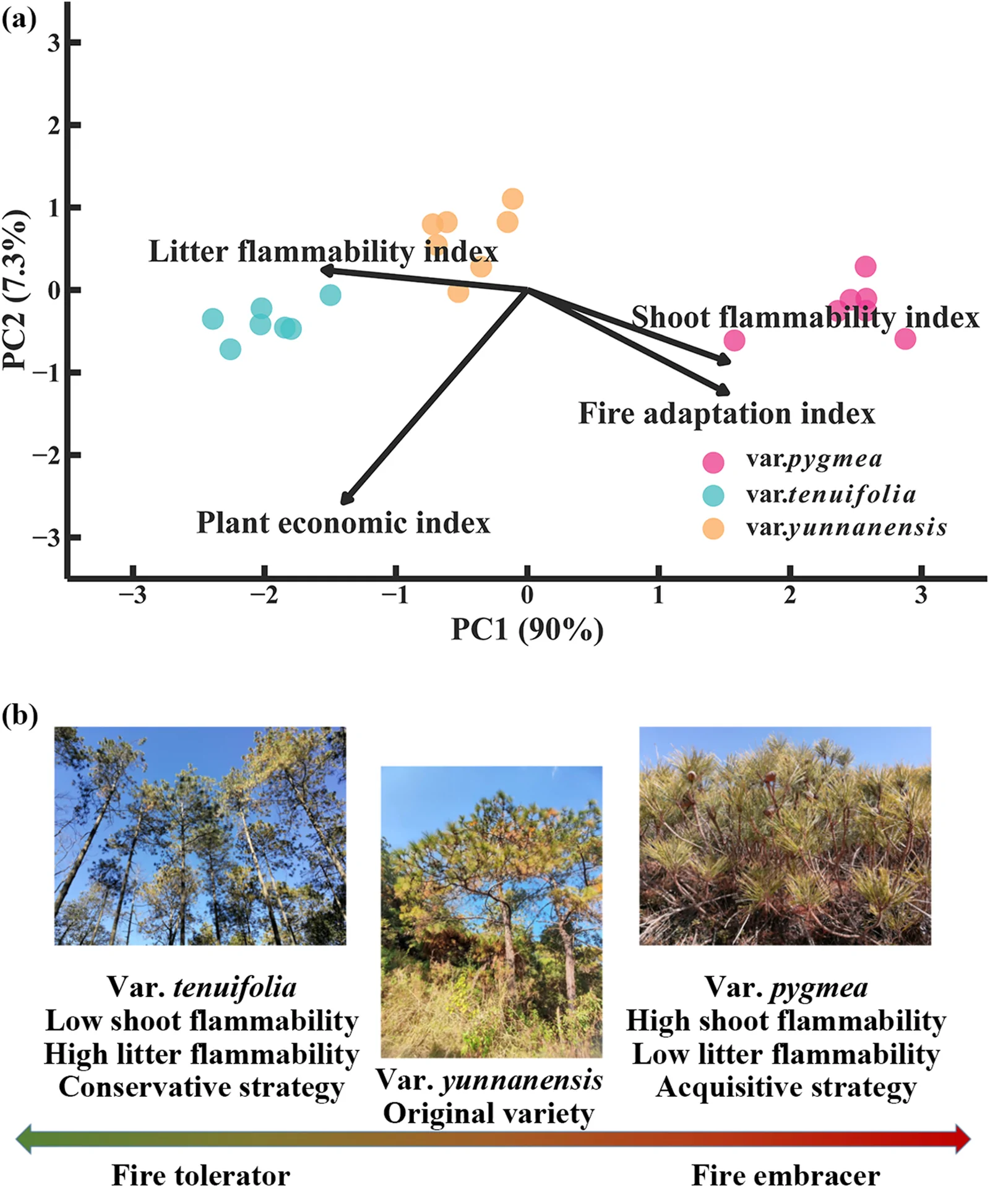
Principal component analysis (PCA) of fire adaptation index, plant economics index, shoot flammability index, and litter flammability index among three varieties. Each point represents a sampling site; colors indicate different varieties (a). Representative photographs of the three varieties illustrating contrasting fire-adaptive strategies: var. *tenuifolia* (left), var. *pygmaea* (right), and var. *yunnanensis* (center). Photos by Xiaohong Chen and Shuting Li (b).

We conducted a principal component analysis using the fire adaptation index, plant economic index, shoot flammability index, and litter flammability index to explore their potential ecological relationships among the different varieties of *Pinus* *yunnanensis* (Fig. 4a). The first two axes explained 90.0% and 7.3% of the total variation, respectively.

Building on the PCA results, we further quantified the pairwise relationships among our four indices (Table 4). The fire adaptation index was very strongly positively correlated with shoot flammability and very strongly negatively correlated with both the litter flammability index and the plant economic index. Likewise, shoot flammability was also highly negatively correlated with the economic index. In contrast, litter flammability and the plant economic index showed a strong positive correlation.

**Table 4:** Correlation coefficients for the fire adaptation index, shoot flammability index, litter flammability index and plant economic index.

	Fire adaptation index	Shoot flammability index	Litter flammability index	Plant economic index
Fire adaptation index	1	**0.94**	**−0.75**	**−0.95**
Shoot flammability index	**0.94**	1	**−0.78**	**−0.93**
Litter flammability index	**−0.75**	**−0.78**	1	**0.84**
Plant economic index	**−0.95**	**−0.93**	**0.84**	1

Note: The indices (Fire adaptation index, Shoot/Litter flammability index, and Plant economic index) represent the scores of the first principal component (PC1) derived from Principal Component Analysis (PCA) performed on the respective group of traits, serving as integrated metrics for each functional category. Values where *P* < 0.05 are shown in bold.

The results suggest that fire adaptation strategies and resource trade-off strategies are intrinsically coupled, var. *pygmaea* employing acquisitive strategy concurrently tend to be “Fire-embracer”, with high shoot flammability and low litter flammability, whereas var. *tenuifolia* employing conservative strategy varieties tend to be “Fire-tolerator”, with low shoot flammability and high litter flammability (Fig. 4b).

## 4 Discussion

The intraspecific variation in fire adaptations observed in *Pinus* *yunnanensis* indicates that some populations have traits to survive surface fires (fire-tolerators) while other are adapted to survive crown fires (fire-embracers). This suggest that *P*. *yunnanensis* is diversifying to occupy different niches defined by the fire regime, indicating that *P*. *yunnanensis* serves as an exemplary case of fire-driven trait divergence within a species. Prior studies that analysed variations in other fire adaptations within *P*. *yunnanensis* support our findings (Tang et al., 2013; Su et al., 2019). For example, the seeds of var. *pygmaea* exhibited the lowest germination rate without heat shock, and heat shock can improve the germination rate greatly (Huo, 2017), further indicating that serotiny may be more important to shrubby populations, while it is not an important trait by which tree-forming varieties survive fire. A post fire study showed that var. *pygmaea* can regenerate quickly after fire by resprouting and seeding recruitment (Zhang et al., 2018), indicating strong post-fire regeneration ability of var. *pygmaea*. The resprouting ability of tree-forming varieties are still unquantified, but it seems to be much weaker than var. *pygmaea* (Zhang et al., 2018; Long, 2022). Nearly all the tree-forming mature individuals can survive ground fires by thick bark and self-pruning (Jin and Peng, 2004), suggesting that tolerance serves as the predominant survival strategy for these tree individuals. All the known facts suggest that *P*. *yunnanensis* is one of the few pines that can survive wildfires as either a fire-tolerator or fire-embracer, diverging into distinct adaptive strategies: the specialized fire-tolerator (var. *tenuifolia*) adapted to surface fires, the fire-embracer (var. *pygmaea*) adapted to crown fires, and the generalized form (var. *yunnanensis*) retaining intermediate traits suited to variable fire regimes.

Flammability plays a crucial role in the fire survival strategy of plants (Pausas et al., 2012; Cui et al., 2020; Kane et al., 2022). Pausas (2015a) posited that the litter flammability of fire-tolerators is high, tree flammability is low; while fire-embracers has opposite flammability traits, due to the fact that fire-tolerators experienced frequent surface fires while fire-embracers usually experience crown fires. However, the evidence supporting this pattern of variation in flammability characteristics is limited. Our study provided micro-level evidence supporting the notion that changes in flammability conferred fitness to plants and is consistent with the survival strategy of plants in fire-prone environments. In this study, we found that var. *pygmaea* exhibited increased shoot flammability due to its shorter needles and the lower leaf moisture content among the varieties. The short leaves of var. *pygmaea* formed densely packed litter, making the flammability of litters lower than that of tree-forming individuals (Scarff and Westoby, 2006; Curt et al., 2011; Cornwell et al., 2015), even if the material itself is highly flammable. Although chemical components can exert an impact on litter flammability (Curt et al., 2011; Grootemaat et al., 2015), packing ratio is a much more crucial factor influencing the flammability of litters (Burton et al., 2021; Zhang et al., 2023). A more loosely packed litter enhances oxygen availability between litter particles, allowing the thermal radiation from the flame to extend further ahead of the fire front (Pastor et al., 2003), increasing the rate of fire spread by bringing a larger area of unburned litter particles close to the combustion temperature (Hoffmann et al., 2012). The lowly flammable shoots and highly flammable litters of var *tenuifolia* prevent the accumulation of understory fuel and restricted potential high-intensity fires (Fonda, 2001; Pausas, 2015a). While in var. *pygmaea*, high shoot flammability confers benefits to individuals by facilitating seeds releasing and increasing the mortality rate of neighboring competitors and facilitating their progeny to dominate more space.

Investigation into the interplay across plant economic traits, fire adaptations, and plant flammability can elucidate the intricate strategies employed by plants to balance resource acquisition with their requirements in order to better co-exist wildfire. However, such studies have been scarcely reported. To our knowledge, our research represents the inaugural exploration of the interplay among these traits. Var. *pygmaea*, the fire-embracer variety, exhibits a more acquisitive strategy than fire-tolerators, characterized by quicker growth rate, higher net photosynthetic rate and lower leaf mass per area (Westoby and Wright, 2006; Reich, 2014). This strategy aligns with the need for quick reproduction and recruitment in habitats where fire is frequent. The conjunction of high shoot flammability, high post-fire regeneration ability and a more acquisitive strategy of var. *pygmaea* can be viewed as an evolutionary response to recurrent crown fires, allowing for rapid reestablishment and providing a competitive advantage to neighbors (Clarke et al., 2015; Pausas, 2015a). In contrast, var. *tenuifolia*, the fire-tolerator, embodies a more conservative PES strategy, reflected in its higher leaf mass per area, conservative gas exchange and delayed sexual maturity. This strategy prioritizes long-term survival and stress resistance over rapid growth (Adler et al., 2014; Kunstler et al., 2016). The thick bark and elevated canopy, in conjunction with low shoot flammability and high litter flammability, ensured the survival of the variety in a fire-prone environment by limiting the accumulation of surface fuel, gaping the surface and crown fuels, and protecting them from heat (Pausas and Keeley, 2009; Lamont et al., 2020).

The high phenotypic variation and fire-prone habitat make *Pinus* *yunnanensis* an ideal species for studying the evolutionary processes that underpin plant adaptation to wildfires. Our study has revealed the varying survival strategies within *P*. *yunnanensis*. Nevertheless, the nuanced trait divergences resulting from diverse fire regimes and how survival strategies are developed by plants to co-exist with wildfires are still unclear. Genetic analysis and identifying genes that effects fire-adaptations will greatly enhance our understanding of fire ecology. Regrettably, such studies are still scarce. Moreover, many flammability-related traits, such as chemical and structural characteristics, are also adapted to other selective pressures (e.g. herbivory and drought). Understanding the proportion of variability in these traits that is driven by recurrent fires is another challenge (Pausas et al., 2017). Substantial further research is required to fully grasp the evolutionary dynamics of fire-related traits in response to varying fire regimes.

## 5 Conclusions

Our study unveils the intricate survival strategies of *Pinus* *yunnanensis* within fire-prone environments, highlighting its capacity to adapt fire both surface and crown fires. Var. *pygmaea*, the fire-embracer, exhibits an acquisitive strategy with heightened shoot flammability and rapid post-fire regeneration, enabling it to quickly regenerate after crown fire. In contrast, var. *tenuifolia*, the fire-tolerator, displays a conservative strategy with lower shoot flammability and higher litter flammability, enhancing its resilience to surface fire through resource conservation and long-term survival. These findings demonstrate how plants evolve diverse flammability traits aligned with their fire adaptation strategies and growth-survival trade-offs. Understanding such strategies and the organ-specific variation in flammability provides insights into wildfire management and the evolutionary mechanisms of plant flammability, guiding future studies in fire-prone ecosystems.

## CRediT authorship contribution statement

**Dachuan Dai:** Investigation, Methodology, Formal analysis, Writing – Original Draft, Writing – Review & Editing. **Shuting Li:** Investigation, Methodology, Formal analysis, Writing – Original Draft, Writing – Review & Editing. **Ying Liu:** Investigation. **Zihan Zhang:** Investigation. **Lihua Tu:** Writing – Review & Editing. **Congde Huang:** Writing – Review & Editing. **Shixing Zhou:** Writing – Review & Editing. **Junxi Hu:** Writing – Review & Editing. **Yang Liu:** Writing – Review & Editing. **Xinhua He:** Writing – Review & Editing. **Juli G. Pausas:** Writing – Review & Editing. **Xinglei Cui:** Conceptualization, Investigation, Writing – Original Draft, Writing – Review & Editing. All authors contributed critically to the drafts, read and approved the final manuscript, and consented to its submission.

## Data availability statement

The data that support the findings of this study are openly available in the Science Data Bank at https://doi.org/10.57760/sciencedb.33467.

## Declaration of competing interest

The authors declare that they have no known competing financial interests or personal relationships that could have appeared to influence the work reported in this paper.
