# Supplementary material for: Fire-embracers possess higher shoot flammability, lower litter flammability and a more acquisitive strategy than fire-tolerators in Pinus yunnanensis
Source: Plant Divers. 2026 Apr 10;48(5):1011–22. doi: 10.1016/j.pld.2026.03.013 (PMC13615787; doi:10.1016/j.pld.2026.03.013)
Supplement: Supplementary file 1 [file mmc1.docx]

**Table S1** Variation in environmental factors among different *P. yunnanensis* varieties.

| Variables | Var. *pygmaea* | Var. *yunnanensis* | Var. *tenuifolia* |
| --- | --- | --- | --- |
| Mean annual temperature (℃) | 15.02 ± 1.10b | 15.85 ± 2.20b | 18.00 ± 1.76a |
| Mean annual precipitation (mm) | 898 ± 75a | 869 ± 171a | 1272 ± 49a |
| Fire activity index | 0.66 ±0.00a | 0.64 ±0.10a | 0.66 ±0.06a |

Note: Populations with the same letter code are not significantly different, based on Fisher's Least Significant Difference (*P* < 0.05). *

**Table S2** Variation in soil nutrient content among different *P. yunnanensis* varieties.

| Variables | Var. *pygmaea* | Var. *yunnanensis* | Var. *tenuifolia* |
| --- | --- | --- | --- |
| Soil total nitrogen content (mg g^-1^) | 0.54 ± 0.09a | 0.45 ± 0.08a | 0.50 ± 0.14a |
| Soil total phosphorus content (mg g^-1^) | 0.47 ± 0.11a | 0.46 ± 0.08a | 0.44 ± 0.10a |

Note: Populations with the same letter code are not significantly different, based on Fisher's Least Significant Difference (*P* < 0.05). *
